# Complications of Reamer–Irrigator–Aspirator System in Pediatric Orthopedic Surgery–Case Series and Scoping Review

**DOI:** 10.3390/children12060700

**Published:** 2025-05-29

**Authors:** Michael William Stickels, Kyung Min Roh, Meghana Belthur, Mohan V. Belthur

**Affiliations:** 1 Department of Orthopedic Surgery, University of Arizona College of Medicine–Phoenix, 475 N 5th St., Phoenix, AZ 85004, USA; kroh@arizona.edu; 2 School of Life Sciences, Arizona State University, 411 N Central Ave., Phoenix, AZ 85004, USA; mbelthur@asu.edu; 3 Department of Orthopedics, Phoenix Children’s Hospital, 1919 E Thomas Rd., Phoenix, AZ 85016, USA; mbelthur@phoenixchildrens.com

**Keywords:** reamer–irrigator–aspirator, RIA, complications, hardware failure, pediatrics, orthopedics

## Abstract

Background: Reamer–irrigator–aspirators (RIAs) are newer orthopedic devices intended to harvest bone while minimizing complications associated with traditional harvesting techniques. Its high success rate has resulted in relatively few studies on its intraoperative and postoperative complications, especially in pediatric populations. This study provides a scoping review of complications associated with the RIA and presents an institutional case series of RIA complications. Materials and Methods: The scoping review was conducted via modified Joanna Briggs Institute (JBI) guidelines. Cases at a single institution were selected on an individual basis as they occurred during or after RIA-associated surgeries. Results: Our case series consists of three males and one female, ranging from 8 to 14 years old, with varying comorbid orthopedic conditions. All complications occurred intraoperatively, with three instances of retained hardware and one instance of cortical disruption. Each complication was classified as stage I according to the modified Clavien–Dindo–Sink classification system. There were no long-term sequelae despite limited management. A scoping review of the literature revealed extremely limited data on pediatric complications, but several mechanical and clinical complications have been described. Conclusions: Complications associated with RIA use in children appear to be inconsequential, but data are very sparse, and further studies are required.

## 1. Introduction

The reamer–irrigator–aspirator (RIA) system (Synthes, Inc., West Chester, PA, USA) is a surgical device introduced for human use, specifically before the implantation of an intramedullary nail, in the early 2000s [1,2]. It was originally designed as an alternative to traditional long bone intramedullary reaming systems to mitigate intraoperative complications [3,4]. However, upon its observed safety and utility in operative practice, it has since been used for other applications, including harvesting osseous autografts, IM reaming in the presence of metastatic disease, and irrigation and debridement of bone for musculoskeletal infection [4,5]. This versatility has led to increased utilization over the past two decades.

The RIA system comprises a single-use reamer head, drive shaft, irrigation/aspiration tubing, a graft harvesting filter, and containment tubing [6]. The irrigation function of the RIA system is designed to flush the intramedullary canal, aiming to maximize thermal energy dispersion and mobilize reamed osseous debris for removal [7,8,9]. The aspiration function of the RIA system uses vacuum-like suction to reduce intramedullary pressure and immediately remove debris from the intramedullary canal [7,8]. Concurrent irrigation and aspiration augment the reaming process by regulating thermogenic and barometric parameters, theoretically reducing complication rates [10].

Complications associated with intramedullary reaming include blood loss, iatrogenic fracture, cortical perforations, thermal injury, and pulmonary fat embolism syndrome (FES) [2,4,11]. Traditional reaming systems are prone to inflicting thermal injury due to less consistent irrigation and the potential for dull blades from instrument reuse; some systems can reach temperatures reaching over 59 degrees Celsius [12]. IM reaming of any kind is still associated with inherent risks and mechanical complications, as RIAs have been demonstrated to have significant complications, including cortical perforation, significant intraoperative blood loss, and retained hardware from device malfunction [2,6,11,13,14]. Studies have demonstrated variable complication rates with RIA use, ranging from 1.7% up to 31% [7,11,15]. The high success rate and safe adverse effect profile of the system have led to a relative scarcity of detailed complication-focused studies, particularly in skeletally immature populations.

Given the distinct anatomical and biomechanical characteristics of children, it remains unclear whether complication rates observed in adults using RIAs can be extrapolated and applied to pediatric populations. Pediatric patients present with unique physiological considerations, including narrower intramedullary canals, lower cortex mineralization content, and ongoing skeletal growth [16,17,18]. Additionally, the biomechanical properties of native osseous structures are much different from those in adults, with pediatric bones having lower yield stress and elastic modulus values but higher strain resistance [16,17]. Additionally, skeletally immature bones have less favorable biomechanical profiles in the setting of torsional deformity [19]. These differences may influence the risk profile of RIA use when compared to adult populations. Despite this, there are few studies analyzing RIA-associated complications in pediatric patients.

With the increasing use of RIAs across a large range of orthopedic indications and extension into pediatrics, there is a notable gap in the literature regarding their complication profile in children. This study aims to address this gap in the literature by providing a scoping review of RIA-associated complications in children and presenting an institutional case series of four pediatric patients who experienced intraoperative or postoperative complications associated with RIA use.

## 2. Materials and Methods

### 2.1. Scoping Review

This review was conducted via an a priori protocol [20]. Although this study intended to focus on complications specifically in the pediatric population, both adult and pediatric studies were included in the final review due to the extremely limited sources of pediatric data. Pediatric-specific data were extracted from mixed studies when available.

#### 2.1.1. Search Strategy and Data Management

A preliminary search of MEDLINE and the COCHRANE Database of Systematic Reviews revealed no existing or ongoing scoping reviews centered around pediatric RIA complications. We selected MEDLINE (via PubMed), Embase (via Elsevier), and COCHRANE Central as our primary databases for the systematic literature search, which was conducted on 13 March 2025. The central search query was individually tailored to the indexing structure of each database; detailed search strategies are provided in Appendix A. Each query attempted to capture discussion or descriptions of RIA complications in pediatric populations. If no results were yielded in a search query, the pediatric filter was removed and the search was re-run in the hopes of gathering more information for review.

#### 2.1.2. Study/Source of Evidence Selection

**Figure 1:**
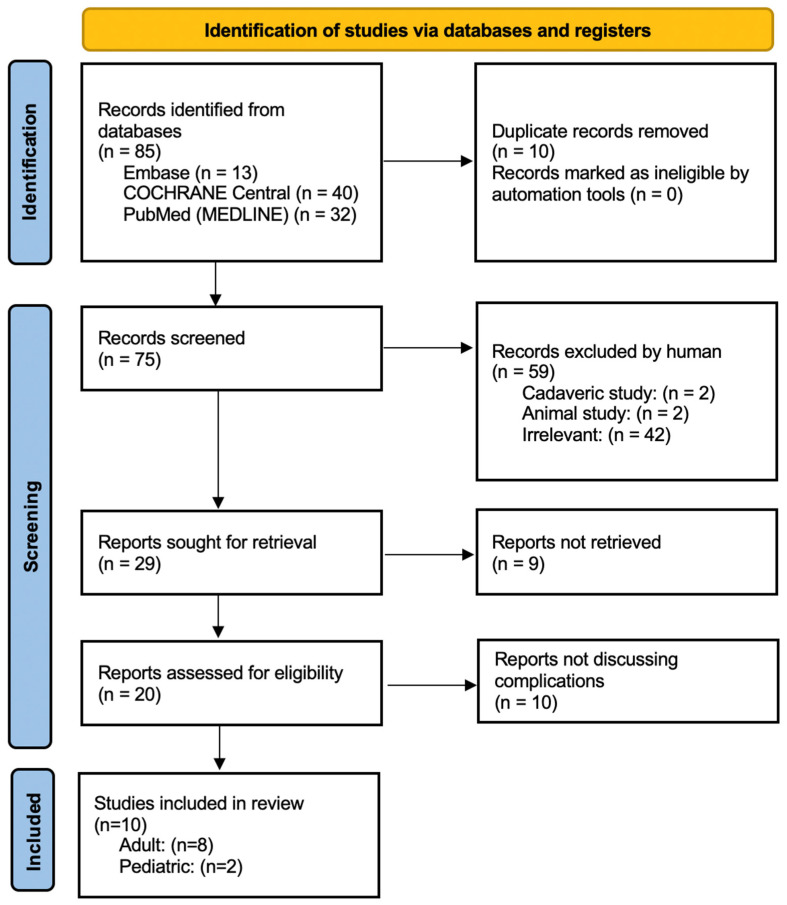
PRISMA 2020 flow diagram illustrating the study selection process for the review of RIA-associated complications in pediatric and adult populations. Databases searched: PubMed (MEDLINE), Embase, COCHRANE Central. Adapted from Page MJ et al. [22].

All identified records were imported into the Rayyan software (version 1.6.0) for organization and duplicate removal [21]. Following a pilot test, titles and abstracts were screened by three independent reviewers (MS, KMR, and MB) to assess eligibility based on the predefined inclusion criteria. Potentially relevant sources were retrieved in full, and citation details were imported into the Zotero reference manager. Full-text articles were independently assessed by two reviewers (MS and MB) to confirm inclusion. The titles of reference lists of included articles were screened for relevancy and potential inclusion in this study. Discrepancies at any stage of the selection process were resolved by discussion or with input from an additional reviewer. The study selection process and results are reported in full and presented in Figure 1, adhering to PRISMA guidelines [22].

#### 2.1.3. Inclusion and Exclusion Criteria

Major inclusion criteria for our study included any form of objective data of immediate or postoperative complications reported for the RIA system in vivo. Studies that utilized cadavers or animals, were in languages besides English, or that were published before 2000 (the year of initial RIA use in humans) were excluded from this study. Additionally, studies not specifically discussing interventional complications of RIA use were excluded. Further, studies were stratified into adult or pediatric studies based on the presence of data that was granular enough to extract.

#### 2.1.4. Types of Sources

This scoping review will consider experimental study designs, including randomized controlled trials, non-randomized controlled trials, before-and-after studies, and interrupted time-series studies. In addition, analytical observational studies, including prospective and retrospective cohort studies, case–control studies, and analytical cross-sectional studies, will be considered for inclusion. This review will also consider descriptive observational study designs, including case series, individual case reports, and descriptive cross-sectional studies for inclusion. Qualitative studies and opinion papers will also be considered that focus on discussing complications related to the RIA system.

#### 2.1.5. Data Charting

Given the focused nature of this review, the extremely limited data around complications in the pediatric population, and its primary role in supporting our case series, the scoping review data from included studies were summarized narratively and thematically without the use of a formal data extraction chart.

### 2.2. Case Series Search Criteria

#### 2.2.1. Search Criteria

Each case was selected for study inclusion on an individual basis as it occurred. Four patients were identified in a pediatric tertiary care center who sustained intraoperative or postoperative complications directly associated with the RIA system. Individual patient charts were analyzed to verify the presence of a documented complication associated with the RIA system, and relevant operative, radiological, and demographic data were extracted.

#### 2.2.2. Inclusion and Exclusion Criteria

Cases were selected on an individual basis according to recorded accounts of the procedure and verified with operative notes and radiography. Cases were disregarded if they did not involve a complication directly related to any RIAs or any plausibly related adjacent intraoperative or postoperative complication.

## 3. Results

### 3.1. Case Series Overview

Our case series consists of four pediatric patients ranging from 8 to 14 years (three males, one female) and four individual long bones (three femurs and one tibia) with surgeries taking place between April 2022 and November 2024. In this same time period, there were a total of 12 children who used RIAs for various indications, yielding an overall complication rate of 33.3%. Specific surgical indications for RIA use among the complication group included bone graft harvest for nonunion correction, irrigation and debridement for osteomyelitis, and assisting in implant cement removal (Table 1). All procedures utilized continuous irrigation and aspiration with the smallest reamer head available (10.5 mm) on varying native intramedullary widths (Table 2). Each patient individually experienced one complication, including retained RIA hardware (n = 3) and a cortical breach (n = 1). Each complication was classified as grade I according to the modified Clavien–Dindo–Sink classification for intervention-associated complications, meaning there was no significant deviation from the intended postoperative recovery course [23]. No patient experienced any negative long-term sequelae at the latest follow-up based on clinical and radiographic evidence (mean follow-up = 394 days) (Table 3). More specific narrative-style case descriptions are included below.

### 3.2. Case Descriptions

**Figure 2:**
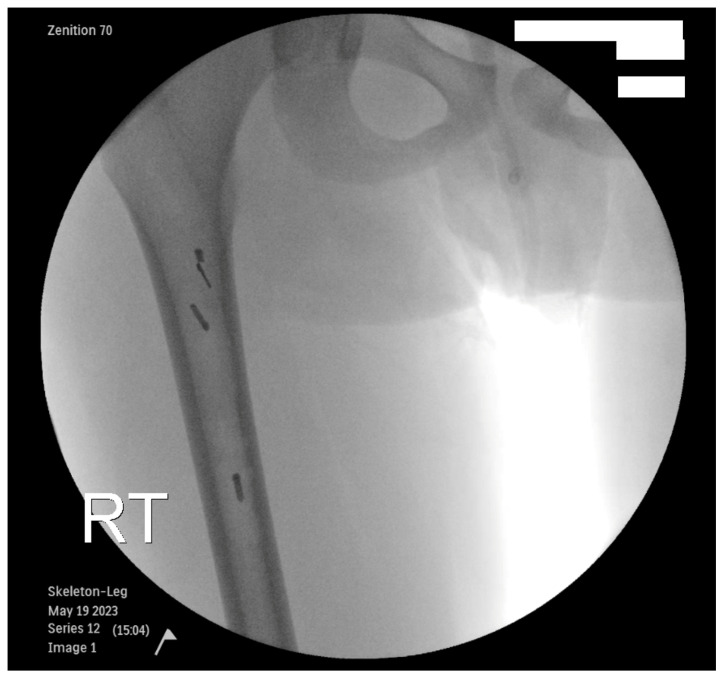
Intraoperative fluoroscopic visualization of four dislodged RIA metal flanges, evidenced by the four hyperdense objects in the femoral intramedullary canal proximal to the isthmus.

Case 1: A 12-year-old male with a history of congenital left femoral discrepancy and prior femoral lengthening complicated by a 5 cm nonunion underwent contralateral femoral bone graft harvest with the RIA system. After initial pre-reaming with a non-aspirator system to the central aspect of the femoral isthmus, the RIA device was inserted for autograft harvest, and severe resistance to progression was felt. The device was retracted, but five metal flanges from the RIA device were retained in the canal as evidenced by immediate intraoperative fluoroscopy (Figure 2). Three pieces were immediately retrieved using a curette and a guide wire, and the remainder were too difficult to extract and were left intraosseous. No complications occurred during follow-up, and the patient remained asymptomatic.

Case 2: A 13-year-old female with chronic methicillin-resistant *Staphylococcus aureus* (MRSA) osteomyelitis of the right tibia underwent irrigation and debridement with the RIA system before the placement of an antibiotic-coated IM nail for prophylactic fracture prevention. During tibial canal preparation, resistance was felt in the distal tibial intramedullary canal, and four reamer flanges were dislodged. One was removed with a pituitary rongeur, and the remainder fell into the distal canal and were left in place; it was determined that the morbidity associated with an additional incision to extract the fragments outweighed the risk of retaining the hardware. No long-term complications were observed.

**Figure 3:**
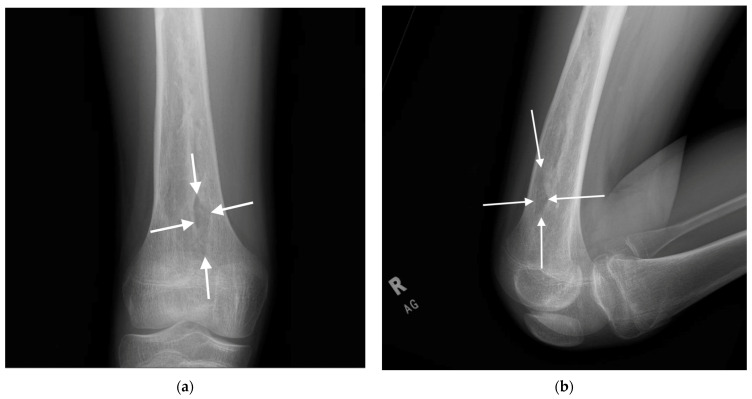
Radiographs of the right anterior distal femoral diaphysis taken seven months postoperatively. White arrows denote the outer margins of the cortical perforation. (**a**) Anteroposterior (AP) radiographs. (**b**) Lateral radiographs.

Case 3: A 14-year-old male with chronic multifocal osteomyelitis underwent femoral nail removal and placement of antibiotic-eluting beads. During reaming to clear the infected bone, the surgeon noted that the femoral canal felt unusually soft, and a subsequent cortical breach was observed with fluoroscopy at the anterior distal femur (Figure 3). No intraoperative intervention was performed, and the patient remained asymptomatic with no functional impairment on follow-up.

Case 4: An 8-year-old male with a prior femoral shaft fracture and open reduction/internal fixation presented with nonunion. During reaming for revision fixation and autograft placement, resistance was encountered, and the RIA device was removed from the intramedullary canal with missing parts. Subsequent fluoroscopy revealed metallic debris from the RIA system, but it was not removed. The patient recovered uneventfully with no functional deficits or symptoms.

## 4. Scoping Review and Discussion

### 4.1. Scoping Review

#### 4.1.1. Clinical Complications

Clinical complications of RIA use refer to patient-centered adverse outcomes. The most feared clinical complication associated with IM reaming systems, including RIA, is fat embolism syndrome [1,24]. FES results from elevated IM pressures during reaming, leading to marrow and fat intravasation into the systemic circulation [24]. Although the RIA system was developed to mitigate this risk through simultaneous aspiration and irrigation, embolic events can still occur, likely due to localized surges in canal pressure [3]. Reported incidence rates in adults range from 0% to 10%, depending on fracture severity, surgical technique, and study size [1,24]. Hall et al. demonstrated a lower embolus score after RIA use in comparison to traditional reaming methods, but the effect of RIA on physiological changes was unclear in their study [24]. The diagnosis of FES remains primarily clinical, often supported by transesophageal echocardiography findings demonstrating right heart strain or characteristic radiographic signs like bilateral pulmonary infiltrates [24]. Management is typically supportive, with oxygen supplementation and critical care monitoring, although mortality rates remain high at around 30% [24,25].

One of the two relevant pediatric studies from our search strategy, a case report by Jacobson et al., documented the success of surgical management of a 14-year-old boy who underwent bilateral femoral IM nailing with RIA utilization for bilateral femoral shaft fractures [1]. Given the patient’s high risk of FES from their injury characteristics, an RIA was utilized bilaterally, and the patient underwent a complete recovery without any complications. The other included a mixed pediatric and adult study by Desai et al., a case series of nine patients undergoing RIA autograft harvesting for nonunion repair, including one 17-year-old male [26]. No complications were recorded during the patient’s procedure or the entire study, providing limited but promising data for pediatric complications with RIAs.

Other potential complications associated with RIA use are iatrogenic fractures or cortical violations. Cortical perforation typically results from eccentric reaming, off-axis torsional loads, excessive reamer sizing, or anatomical deformities [2,6]. Pediatric patients are particularly vulnerable due to narrower canals and skeletal immaturity [1,18,26]. While most cortical breaches are identified intraoperatively through fluoroscopic imaging and remain asymptomatic when small, larger perforations may require postoperative activity modification or additional stabilization [2]. For this reason, surgeons must consider the diameter of the intramedullary canal preoperatively, especially in skeletally immature individuals. Reaming with too large a diameter in comparison to the intramedullary canal can lead to excessive cortical thinning or an outright iatrogenic fracture [2,4,7,8,13,27,28].

Intraoperative hemorrhage is another important consideration during RIA use. Several studies have demonstrated a significant association with clinically significant blood loss after RIA autograft harvests [11,29,30]. Although continuous irrigation in the RIA system reduces thermal necrosis compared to traditional reamers, the high flow rate may potentiate blood loss from intramedullary vessels by disrupting coagulation [11]. Additionally, postoperative pain and infection remain recognized, albeit relatively uncommon, complications. Compared to iliac crest bone graft harvesting, RIAs appear to be associated with lower donor site pain and serve as a favorable alternative for autograft harvesting [2,28,31]. Infection rates are reported to be lower than in traditional reaming systems [11,28].

#### 4.1.2. Mechanical Failures

Mechanical failures encompass device-related malfunctions specifically related to the RIA system, independent of direct patient tissue injury. Retained hardware fragments are among the most frequently reported mechanical failures for the RIA system, although the presence of mechanical complications is relatively rare [6]. In our case series, three of the four complications involved retained metallic debris (two with RIA flanges, one with miscellaneous debris), all associated with increased resistance during reaming. Factors implicated in hardware retention include off-axis reaming forces, narrow canals, anatomical deformities, improper device assembly, and repeated use of single-use heads [6,30,32]. Retrieval strategies vary depending on fragment location, with some cases requiring intraoperative extraction and others managed expectantly if the risk of removal outweighed the potential benefit [6,30,33,34].

The management of retained hardware in our case series was partially successful with the use of a pituitary rongeur, a long laparoscopic grasping forceps, and a curette, but was not successful for removing debris that had migrated distally in the intramedullary canal. A surgical technique guide by Chloros et al. states that an optimal method for metallic debris extraction involves the use of a nail extraction hook [6]. Other reported difficulties include head–driveshaft dissociation, driveshaft breakage, and autograft capture system disengagement [6,35,36]. Most documented mechanical complications are clinically inconsequential.

### 4.2. Discussion

All observed complications at our institution occurred intraoperatively and were mechanical in nature. Three cases involved retained hardware or device debris, and one involved cortical perforation. In each case, the complication was at least partially addressed immediately or managed conservatively without resulting in long-term sequelae. All complications were detected intraoperatively through fluoroscopy. Despite procedural and anatomic deviations, outcomes were uniformly favorable across all cases.

In our case series, all retained hardware events were immediately preceded by an abnormal sensation of increased resistance with the forward reaming motion. This increased resistance and subsequent hardware malfunction indicate a potential irregularity of the canal, improper angle of reamer entry, eccentric reaming, or excessive off-axis torsional load on the cortex. The potential for these technical mechanical errors, combined with the presence of an underlying cortical or angular deformity in some of our patients, could potentially increase the risk of structural device malfunction or cortical perforation. While the greater trochanteric entry point is preferred in pediatric femoral antegrade nailing to reduce the risk of avascular necrosis and growth plate injury, it may alter the reaming trajectory and contribute to eccentric reaming, mechanical strain, and potential malfunction of the RIA system [6,14,15,16,30,32,35,36]. Further, skeletally immature individuals are more likely to have higher degrees of physiologic anterior femoral bowing, creating a non-linear femoral canal that is inherently more susceptible to off-trajectory reaming complications [37]. These anatomical and technical differences may plausibly explain the relatively high mechanical complication rate seen in our case series in comparison to adult studies. Although each patient had a favorable recovery and prognosis, this high complication rate must be noted.

Our study contributes several novel findings to the sparse literature on RIA use in pediatric populations. First, we add four new cases of intraoperative RIA-associated complications in skeletally immature patients, enriching the limited data pool. Second, our combined case series and scoping review approach offers a broader view of RIA complication profiles by integrating pediatric-specific observations with findings from larger adult studies. Of the included studies, only four mentioned pediatric patients, and only two reported pediatric complications with sufficient detail to be extractable. Thus, our case series substantially expands the available pediatric dataset from two to six documented cases. However, the lack of data is the main source of limitations for our study. Our findings contribute to the literature and help build the complication profile of the RIA system in children, but our limited data gives us little power to provide clinical recommendations. Additionally, our case series may reflect intrinsically higher mechanical complication rates due to the previously discussed anatomical factors specific to skeletally immature individuals [14,15,16,33,34]. Additionally, the limited data made the scope of the paper rather narrow; the pivot to adult-based RIA complications contextualized in pediatrics was our solution to this.

Based on our findings, surgeons using the RIA system in pediatric patients should maintain a low threshold for intraoperative fluoroscopic evaluation, particularly when abnormal resistance is encountered during reaming. Given the gravity of orthopedic intervention in skeletally immature individuals, meticulous surgical planning, intraoperative vigilance, and proper device handling should be emphasized. Surgeons should be prepared to manage hardware-related failures within the intramedullary canal with laparoscopic grasping forceps or specialized nail extraction devices. Further evaluation is required to characterize the complication profile of the RIA system in pediatrics.

## 5. Conclusions

The RIA is a safe surgical device with increasing applications in pediatric orthopedic surgery. Although it has been associated with significantly morbid complications in the adult population, the limited data available for pediatric cases suggest that its use in children is safe and well tolerated.

## Figures and Tables

**Table 1:** Shows the basic demographic information and prior medical history of each patient in our cohort who sustained RIA-associated complications.

Patient Characteristics							
Case	Age	Sex	BMI	Prior Orthopedic Conditions	Medical PMH	Prior Orthopedic Surgeries	Surgical Indication
1	12	M	15.63	Congenital left femoral limb length discrepancy, contralateral genu valgum, left femoral cortex nonunion	Asthma	5 cm left femoral lengthening with IM magnetic nail, distal medial femur physeal tethering	Nonunion
2	13	F	21.47	Chronic MRSA osteomyelitis of the left proximal tibial metaphysis, subperiosteal abscess	None	None	Osteomyelitis
3	14	M	14.95	Chronic multifocal (autoimmune) osteomyelitis, subperiosteal abscess, Brodie’s abscess	Disease-modifying rheumatologics	Brodie’s abscess, right femur I&D, subperiosteal abscess biopsy, right femoral IM antibiotic nail	Retained hardware
4	8	M	28.14	Traumatic fracture of the left femoral diaphysis	None	ORIF with flexible nails 8 months prior with I&D, tenodesis of the semitendinosus	Nonunion

**Table 2:** Shows specific surgical details and reamer–irrigator–aspirator parameters during the surgery. Aspirate volumes were not recorded for non-autograft procedures.

Surgical Parameters												
Case	Bone	Side	RIA Procedure	Ipselateral Deformity	Entry Point	Guide Pin	Medulla Width	Pre-Ream Size	RIA Size	Aspiration	Irrigation	Aspirate Volume
1	Femur	R	Bone harvest for nonunion	None	Greater trochanter	Yes	10 mm	8.5 mm	10.5 mm	Continuous	Continuous	20 cc
2	Tibia	L	Irrigation and debridement	None	Lateral tibial spine	Yes	10 mm	10.5 mm	10.5 mm	Continuous	Continuous	N/A
3	Femur	R	Irrigation and debridement	None	Greater trochanter	No	14 mm	12 mm	10.5 mm	Continuous	Continuous	N/A
4	Femur	L	Bone harvest for nonunion	Nonunion	Greater trochanter	Yes	14.1 mm	10 mm	10.5 mm	Continuous	Continuous	15 cc

**Table 3:** This table summarizes each specific complication sustained, management, and outcomes for each patient.

Complication Description and Outcomes									
Case	Complication	Time	Diagnostic Modality	Severity	Management	Outcome	Follow-Up	Complication Resolution	Corrective Procedure
1	Retained hardware, 5 flanges	Intraop	Fluoroscopy	Grade I	Removal of 3 pieces with a curette	No long-term sequelae	535 days	No	None
2	Retained hardware, 4 flanges	Intraop	Fluoroscopy	Grade I	Removal of 1 piece with pituitary rongeur	No long-term sequelae	248 days	No	None
3	Anterior cortical breach	Intraop	Fluoroscopy	Grade I	Nothing	No long-term sequelae	670 days	No	None
4	Retained hardware, metal debris	Intraop	Fluoroscopy	Grade I	Nothing	No long-term sequelae	123 days	No	None

## Data Availability

The datasets presented in this article are not readily available because of the associated technical and time limitations, in addition to the presence of protected personal health information that is inseparable from the dataset.

## Abbreviations

The following abbreviations are used in this manuscript:

RIA	Reamer–Irrigator–Aspirator
JBI	Joanna Briggs Institute
MRSA	Methicillin-resistant *Staphylococcus aureus*
AP	Anteroposterior
FES	Fat embolism syndrome

## Appendix A

### Appendix A.1. MEDLINE (PubMed) Search Strategy

(((((reamer irrigator aspirator) OR (reamer-irrigator-aspirator)) OR (R.I.A.))) AND (((((complications) OR (complication)) OR (adverse effects)) OR (adverse effects)) OR (morbidity))) AND ((((((((((((pediatric) OR (paediatric)) OR (paediatrics)) OR (pediatrics)) OR (children)) OR (child)) OR (toddler)) OR (youth)) OR (adolescent)) OR (adolescence)) OR (juvenile)) OR (infant)).

### Appendix A.2. COCHRANE Central Search Strategy

(reamer irrigator aspirator) OR (reamer-irrigator-aspirator) OR (R.I.A.). (Word variations have been searched.)

### Appendix A.3. Embase Search Strategy

‘reamer irrigator aspirator’ or ‘reamer irrigator aspirator system’ or ‘reamer-irrigator-aspirator’ or ‘R.I.A.’ AND (‘complication’ or ‘adverse event’ or ‘morbidity’ or ‘complications’ or ‘adverse effects’) AND (‘child’ or ‘pediatric hospital’ or ‘adolescent’ or ‘juvenile’ or ‘toddler’ or ‘infant’ or ‘infant disease’ or ‘childhood disease’ or ‘adolescence’).
